# Supplementary material for: A widespread actinobacterial G protein system regulates production of specialized metabolites in Streptomyces coelicolor
Source: mSphere. 2026 Aug 14;11(9):e00568-25. doi: 10.1128/msphere.00568-25 (PMC13621833; doi:10.1128/msphere.00568-25)
Supplement: Supplementary file 1 [file msphere.00568-25-s0008.pdf]

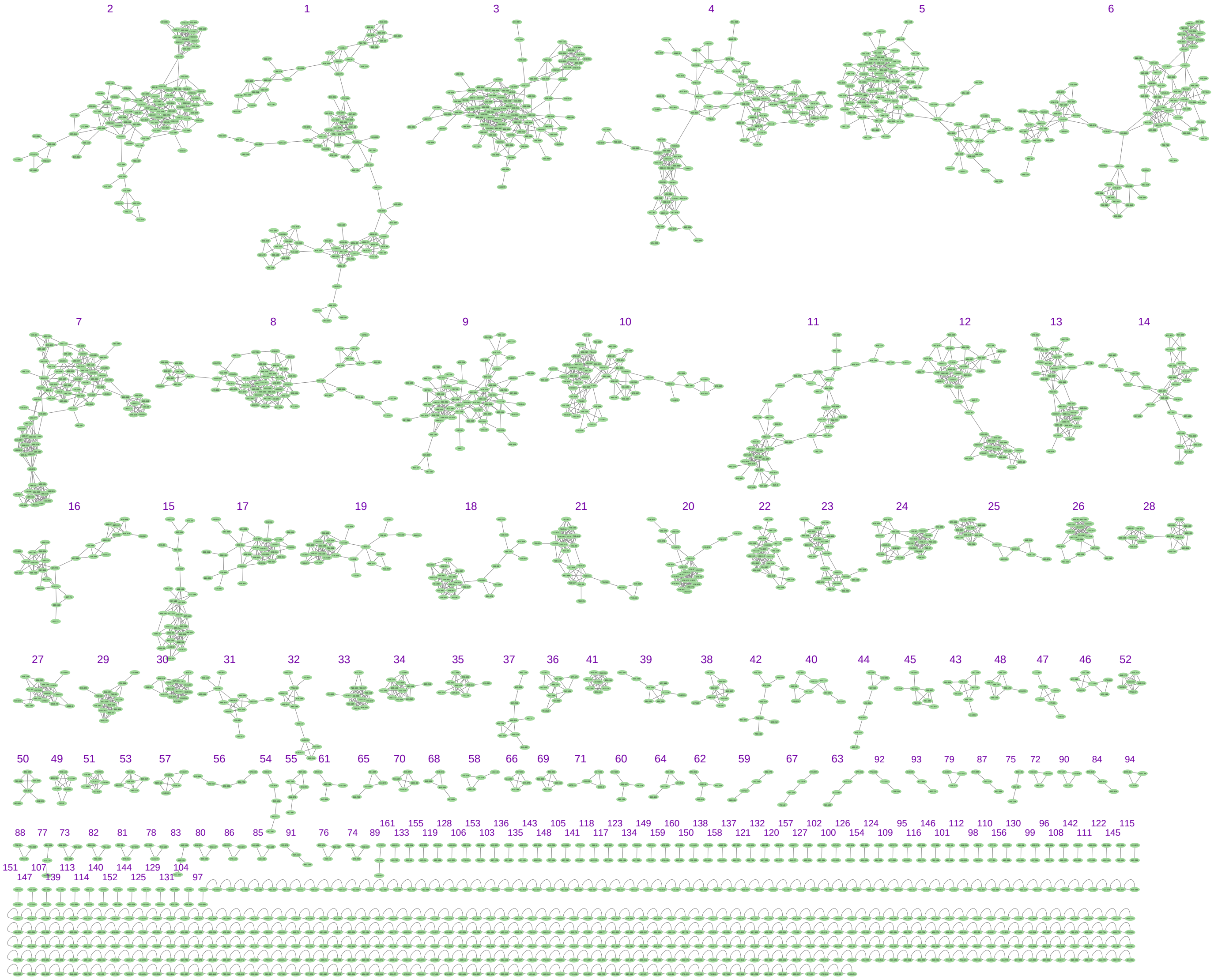
2 1 3 4 5 6
7 8 9 10 11 12 13 14
16 15 17 19 18 21 20 22 23 24 25 26 28
27 29 30 31 32 33 34 35 37 36 41 39 38 42 40 44 45 43 48 47 46 52
50 49 51 53 57 56 54 55 61 65 70 68 58 66 69 71 60 64 62 59 67 63 92 93 79 87 75 72 90 84 94
161 155 128 153 136 143 105 118 123 149 160 138 137 132 157 102 126 124 95 146 112 110 130 96 142 122 115
88 77 73 82 81 78 83 80 86 85 91 76 74 89 133 119 106 103 135 148 141 117 134 159 150 158 121 120 127 100 154 109 116 101 98 156 99 108 111 145
151 107 113 140 144 129 104
147 139 114 152 125 131 97
