# Supplementary material for: A widespread actinobacterial G protein system regulates production of specialized metabolites in Streptomyces coelicolor
Source: mSphere. 2026 Aug 14;11(9):e00568-25. doi: 10.1128/msphere.00568-25 (PMC13621833; doi:10.1128/msphere.00568-25)
Supplement: Supplementary file 2 [file msphere.00568-25-s0009.docx]

**Supplementary Information**

A widespread Actinobacterial G Protein System regulates production of specialized metabolites in *Streptomyces coelicolor*

Luis M. Cantu Morin, Aditya Vunnum, Sydney Binns, and Matthew F. Traxler*

Department of Plant and Microbial Biology, University of California, Berkeley, California, USA

**Contents**

| **Materials and Methods** | 2 |
| --- | --- |
| Genome Mining and Phylogenetic Analysis |  |
| Strain Construction and Growth Conditions |  |
| MALDI Imaging Mass Spectrometry (MALDI-IMS) |  |
| LC-MS/MS Metabolomics of Agar Extracts |  |
| Feature-Based Molecular Networking and Spectral Annotation |  |
|  |  |
| **Supplementary File Legends** | 6 |
| Legends for six accompanying Supplementary Files |  |
|  |  |
| Supplementary Figures | 7 |
| Supplementary Figure 1. Annotated LC-MS/MS spectrum of Amychelin.  Supplementary Figure 2. Annotated LC-MS/MS spectrum of Actinorhodin 3. |  |
| Supplementary Figure 3. Annotated LC-MS/MS spectrum of Actinorhodin 4.  Supplementary Figure 4. Annotated LC-MS/MS spectrum of C11 acyl-DFO.  Supplementary Figure 5. Annotated LC-MS/MS spectrum of C12 acyl-DFO.  Supplementary Figure 6. Annotated LC-MS/MS spectrum of deferoxamine. |  |
| Supplementary Figure 7. Annotated LC-MS/MS spectrum of deferoxamine E (DFO-E)**.** |  |
| Supplementary Figure 8. Annotated LC-MS/MS spectrum of undecylprodigiosin  Supplementary Figure 9. Features from Fig. 2B with Matched Cluster annotation  Supplementary Figure 10. Molecular Networking analysis with annotated features from Fig. 2B. |  |
| **References** | 21 |

**Materials and Methods**

**Genome Mining and Phylogenetic Analysis**

A total of 485 high-quality genomes classified under the phylum Actinomycetota from the ActDES dataset were downloaded from IMG. Protein sequences were clustered into orthogroups using OrthoFinder (v2.5.4)(1) with default parameters. Conservon-associated histidine kinases (*cvnA*) were identified based on orthogroup membership and domain annotations, specifically the presence of HisKA and HATPase_c domains. A gene tree of *cvnA* homologs was inferred using IQ-TREE (v2.2.0)(2), with model selection performed via ModelFinder and branch support assessed using 1,000 ultrafast bootstrap replicates. A species tree of the same 485 genomes was constructed from a concatenated alignment of 120 conserved bacterial marker genes using the same software, and rooted at *Eggerthellales*. The gene and species trees were visualized in R using the ggtree package(3), with additional annotation tracks showing the presence or absence of *cvnA* clade orthologs. The *S. coelicolor* genome encodes thirteen AGPSs, and the different *cvnA* homologs from *S. coelicolor* were used as starting points to define AGPSs families. Specifically, we considered *cvnA* sequences to be in a family if they fell within ten nodes of a *S. coelicolor* *cvnA* homolog.

**Strain Construction and Growth Conditions**

Deletion mutants of *cvnA8,* including *cvnA8*, *cvnF8*, *cvnB8*, *cvnC8*, and *cvnD8*, were generated in *Streptomyces coelicolor* M145 using the REDIRECT PCR-targeting system in our previous work(4). For interaction assays, ISP2 agar was poured into 10 cm Petri dishes to a depth of approximately 3 mm. A total of 0.5 µL of *Amycolatopsis* sp. AA4 spore stock was spotted near the center of each plate. Approximately 2 mm adjacent to the AA4 spot, 0.5 µL of *S. coelicolor* spore stock, either wild-type or mutant, was placed to allow interspecies interaction. An additional 0.5 µL patch of *S. coelicolor* was spotted 2 cm away from the interaction pair to serve as a non-interacting control. Plates were incubated at 30 °C for 3 days prior to mass spectrometry analysis.

**MALDI Imaging Mass Spectrometry (MALDI-IMS)**

Following incubation, a square region of agar containing the interaction zone was excised and carefully transferred onto a standard ground steel MALDI target plate (Bruker or equivalent). The agar was allowed to air-dry for 5 minutes at 30 °C prior to matrix application. Matrix was applied by sublimation using 2,5-dihydroxybenzoic acid (DHB) at 30 mg/mL in 50:50 acetonitrile:water with 0.1% trifluoroacetic acid (TFA), following a modified protocol based on(6).

MALDI imaging was performed using a SubAP/MALDI(ng) ionization source (MassTech, Columbia, MD) coupled to a Thermo Q-Exactive high-resolution mass spectrometer (Thermo Fisher Scientific, San Jose, CA). The SubAP/MALDI(ng) operates under atmospheric pressure and utilizes a laser spot size of <10 µm, equipped with an ion funnel to enhance ion transmission and improve signal-to-noise ratio. Full MS1 scans were acquired in positive ion mode across a mass-to-charge (m/z) range of 100-2000. Laser energy was set between 50-80% with a repetition rate of 1 kHz. Imaging was performed with a pixel size of 10 µm and a laser velocity of 1.5 mm/min on single biological replicates.

Data were processed in R using the Cardinal package(5). Shrunken Centroid Clustering (NSC) was used to identify spatially distinct metabolite patterns across the colony surface. Amychelin, a siderophore produced by *Amycolatopsis* sp. AA4, was detected at m/z 761.2713 ± 0.5 and served as a reference for interaction-specific chemical features.

**LC-MS/MS Metabolomics of Agar Extracts**

Metabolomic profiling of interaction and control conditions was performed using protocols adapted from Pesotti et al. (6). Patches were grown in conditions described above. After 3 days of incubation at 30 °C, agar patches approximately 1 cm² in size were excised. For the non-interacting controls, the patch of *S. coelicolor* was located in the center of the 1 cm² plug. For interacting patches of *S. coelicolor*, the excised plug encompassed the *S. coelicolor* patch, but avoided sampling visible *Amycolatopsis sp*. AA4 biomass. In these plugs, the *S. coelicolor* patch was positioned near the edge of the plug. For each replicate, three agar plugs were extracted in 1 mL of 100% methanol at room temperature with gentle shaking. Extracts were dried under vacuum and resuspended in 500 μL of methanol containing an internal standard (reserpine at 1 μg/mL). Samples were sonicated for 5 minutes and centrifuged for 5 minutes at 13,000 rpm to remove particulate matter.

Metabolite extracts were analyzed in a randomized order using an ultra-high-pressure liquid chromatography system (Thermo Dionex UltiMate 3000, Thermo Fisher Scientific, USA) coupled to a high-resolution tandem mass spectrometer (Thermo Q-Exactive Quadrupole-Orbitrap, Thermo Fisher Scientific) equipped with a heated electrospray ionization source. Chromatographic separation was performed on a Thermo Scientific Acclaim RSLC C18 column (50 mm × 2.1 mm, 2.2 µm particle size). The mobile phases consisted of 0.1% formic acid in water (A) and 0.1% formic acid in acetonitrile (B), delivered at a flow rate of 0.4 mL/min using the following gradient: 0–1 min, 30% B; 1–13 min, 30–100% B; 13–16.5 min, 100% B; 16.5–17 min, 100–30% B; and 17–20 min, 30% B. The injection volume was 5 µL, and the column oven temperature was maintained at 35 °C.

Samples were analyzed in both LC-MS and LC-MS/MS modes using centroid data acquisition. For full-scan MS1 acquisition, the Orbitrap resolution was set to 35,000 full width at half-maximum (FWHM), with an automatic gain control (AGC) target of 1 × 10⁶ ions, a maximum injection time (IT) of 100 ms, and a mass range of m/z 200–1500. For LC-MS/MS, data-dependent acquisition (DDA) was used in Top5 mode to fragment the five most intense precursor ions per cycle. Dynamic exclusion was applied for 5 seconds to prevent redundant fragmentation. MS/MS spectra were acquired at a resolution of 17,500 FWHM with an AGC target of 1 × 10⁵ ions and a maximum IT of 50 ms. Fragmentation was performed using stepped normalized collision energies of 20, 30, and 40, with an isolation window of 3 m/z.

For analysis in Fig. 2B, features were aligned and processed in R using the xcms and MSnbase packages. Only features that appeared in all three replicates were retained. Principal Component Analysis (PCA) was performed using the prcomp function to assess clustering of metabolic profiles across wild-type and mutant strains in interaction and control conditions. Heatmaps of the 100 most variable features were generated using the pheatmap package(8), and clustered using via complete linkage and Euclidean distance.

For heatmaps in Fig. 2C, raw data were processed using MZmine 4.7.8 for feature detection, retention time alignment, and quantification(7), as it allows for ready retrieval and manual annotation of MS2 spectra. Known metabolites, including amychelin and actinorhodin derivatives, were annotated based on accurate mass within 5 ppm error followed by manual matching/annotation of MS2 spectral peaks as shown in SI figures 1-9.

Feature-Based Molecular Networking and Spectral Annotation

Processed LC-MS/MS data were exported directly from MZmine for feature-based molecular networking (FBMN) using the GNPS2 platform(9). MZmine was used to generate the required .mgf (MS/MS spectra) and .csv (feature quantification) files, along with a metadata table containing sample group information (e.g., strain, condition, replicate). All files were uploaded to GNPS via the Feature-Based Molecular Networking workflow. The precursor ion mass tolerance was set to 2 ppm. Edges in the resulting network were filtered to retain only those with a cosine similarity score above 0.7 and at least six matched fragment peaks. Spectral library matching was performed against the GNPS public libraries. Putative annotations were retained if the cosine similarity exceeded 0.7 and the number of shared peaks was more than 6. Spectral matches were manually inspected using GNPS mirror plots, and compound identities were further supported by exact mass and MS/MS fragmentation as annotated and/or comparison to published spectra.

Features in the heatmap shown in Figure 2B were matched to nodes in the molecular network based on MS1 *m/z* values with a mass tolerance of +/-0.005 Dalton. We note that features in Figure 2B were aligned and processed in R using the xcms and MSnbase packages while GNPS was used for node/feature concatenation to produce the molecular networks. These independent processes can yield *m/z* values that potentially vary for the same compound. Thus, the +/-0.005 Dalton represents a stringent cutoff for feature matching across the two outputs. Numbers in the Matched Cluster column in Figure S9 refer to the cluster annotations shown in purple in Figure S10.

**Data availability**

Peak intensities for all features are available in the SI file: features.csv. GNPS feature-based molecular network can be built with the following SI files: README_import_instructions.txt (contains instructions for building network in Cytoscape), edges_corrected.csv and nodes_corrected.csv contain data for network construction in cytoscape.

**Supplementary File Legends**

Eight additional supplementary files are provided.

**Fig_2B_t-test_SI.xlsx :** xls with unpaired t-test *p*-values for features displayed in Fig. 2B. For t-testing, the data for the two conditions (alone or in interactions) were treated separately. Values for the three mutant replicates were compared to values for the WT replicates.

**Fig_2C_features_SI.xlsx :** xls with presence/absence values and unpaired t-test p values. For t-testing, the data for the two conditions (alone or in interactions) were treated separately. Values for the three mutant replicates were compared to values for the WT replicates.

**features.csv** : This csv file contains intensities for all features. Features were aligned and processed in R using the xcms and MSnbase packages.

**README_import_instructions.txt** : This file contains instructions for constructing the molecular network in cytoscape using the two files listed below (nodes_corrected and edges_corrected files).

**nodes_corrected.csv :** This file contains node annotation information for network reconstruction in Cytoscape.

**edges_corrected.csv** : This file contains edge information for network reconstruction in cytoscape.

**whole_network_cluster_ID_overlay_v15.pdf :** This file contains a machine readable and zoomable pdf image of the feature network shown in Figure S10.

**labeled_cluster_node_mz_values_v15.csv :** This file contains the GNPS node IDs, precursor mass, and parent mass for each node assigned to clusters in the network. The pdf_cluster_id contains the cluster ID that corresponds to the IDs shown in purple in Figure S10.

**Supplementary Figures**

Figure S1. Annotated LC-MS/MS spectrum of Amychelin**.** Fragmentation spectrum of the parent ion at m/z 739.2888, detected in positive mode and annotated as Amychelin. Putative fragment ions annotated with an asterisk in red and correspond to known or predicted substructures based on manual annotation and reference literature (10). (B) Molecular structure and related fragments are shown with exact monoisotopic masses and their observed MS2 peaks. These peaks also match peaks found in reference literature (10).

Figure S2. Annotated LC-MS/MS spectrum of Actinorhodin 3**.** Fragmentation spectrum of the parent ion at m/z 633.1239, detected in positive mode and annotated as Actinorhodin 3. Putative fragment ions are highlighted in color and correspond to known or predicted substructures based on manual annotation. Molecular structures of Actinorhodin 3 and related fragments are shown below with exact monoisotopic masses provided.

Figure S3. Annotated LC-MS/MS spectrum of Actinorhodin 4**.** Fragmentation spectrum of the parent ion at m/z 635.1395, detected in positive mode and annotated as Actinorhodin 4. Putative fragment ions are highlighted in color and correspond to known or predicted substructures based on manual annotation. Molecular structures and related fragments are shown below with exact monoisotopic masses provided.

**Figure S4.** Annotated LC-MS/MS spectrum of C11 acyl-DFO**.** (A) Fragmentation spectrum of the parent ion at m/z 701.5165, detected in positive mode and annotated as C11 acyl-DFO. Putative fragment ions annotated with an asterisk in red and correspond to known or predicted substructures based on manual annotation and reference literature(11). (B) Molecular structure and related fragments are shown with exact monoisotopic masses and their observed MS2 peaks.

**Figure S5.** Annotated LC-MS/MS spectrum of C12 acyl-DFO**.** (A) Fragmentation spectrum of the parent ion at m/z 715.5326, detected in positive mode and annotated as C12 acyl-DFO. Putative fragment ions annotated with an asterisk in red and correspond to known or predicted substructures based on manual annotation and reference literature(11). (B) Molecular structure and related fragments are shown with exact monoisotopic masses and their observed MS2 peaks.

Figure S6. Annotated LC-MS/MS spectrum of **deferoxamine**. (A) Fragmentation spectrum of the parent ion at m/z 561.3606, detected in positive mode and annotated as deferoxamine (DFO). Putative fragment ions annotated with an asterisk in red and correspond to known or predicted substructures based on manual annotation and literature (11). (B) Molecular structure and related fragments are shown with exact monoisotopic masses and their observed MS2 peaks. These peaks also match MS2 reference in previous published literature (11).

Figure S7**.** Annotated LC-MS/MS spectrum of deferoxamine E (DFO-E)**.** Fragmentation spectrum of the parent ion at m/z 601.3553, detected in positive mode and annotated as deferoxamine E (DFO-E)**.** Putative fragment ions annotated with an asterisk in red and correspond predicted substructures based on manual annotation. Asterisks in green correspond to peaks matching annoted MS2 spectra found in MassBank of North America (RRID:SCR_015536) with query “exists(compound.metaData.name:'InChIKey' and compound.metaData.value:'NHKCCADZVLTPPO-UHFFFAOYSA-N')” for DFO-E. (B) Molecular structure and related fragments are shown with exact monoisotopic masses and their observed MS2 peaks.

Figure S8. Annotated LC-MS/MS spectrum of undecylprodigiosin. Fragmentation spectrum of the parent ion at m/z 394.2854, detected in positive mode and annotated as undecylprodigiosin. Putative fragment ions annotated with an asterisk in red correspond to known substructures based on reference literature(12) and manual annotation. Fragment ions annotated with green asterisk correspond to ions annotated in reference literature (12). (B) Molecular structure and related fragments are shown with exact monoisotopic masses and their observed MS2 peaks. These peaks also match peaks found in reference literature(12).

**Figure S9. Features from Fig. 2B with Matched Cluster annotation.** This version of Fig. 2B includes a ‘Matched Cluster’ column directly to the right of the heatmap. The dataset was analyzed in parallel using GNPS to generate a feature-based molecular network as described in the Materials and Methods (network shown in Figure S10 below). Features in the heatmap were matched to nodes in the molecular network based on MS1 *m/z* values with a mass tolerance of +/-0.005 Dalton. Numbers in the Matched Cluster column refer to the cluster annotations shown in purple in Figure S10. The top five most common clusters are highlighted with different colors (cluster 1: red, cluster 10: yellow, cluster 27: cyan, cluster 3: orange, and cluster 4: green). All other clusters had fewer than three matches.

**Figure S10. Molecular Networking analysis with annotated features from Fig. 2B.** Undecylprodiginine [M+H] and Acyl-DFOs (11 and 12, both [M+H]+ adducts) group together with other features based on MS2 similarity, suggesting that other features in these groups may share structural components. This network included all samples shown in Fig. 2B, with features found in a methanol control removed. The network was constructed with default parameters (min cosine value of 0.7). Purple cluster annotations were assigned to enable comparisons with features highlighted in Figure S9 above. A pdf version of this network is available in SI file: whole_network_cluster_ID_overlay_v15.

**References**

1.	Emms DM, Kelly S. 2019. OrthoFinder: Phylogenetic orthology inference for comparative genomics. Genome Biol 20.

2.	Nguyen LT, Schmidt HA, Von Haeseler A, Minh BQ. 2015. IQ-TREE: A fast and effective stochastic algorithm for estimating maximum-likelihood phylogenies. Mol Biol Evol 32:268–274.

3.	Yu G, Smith DK, Zhu H, Guan Y, Lam TTY. 2017. ggtree: an r package for visualization and annotation of phylogenetic trees with their covariates and other associated data. Methods Ecol Evol 8:28–36.

4.	Bonet B, Ra Y, Cantu Morin LM, Soto Bustos J, Livny J, Traxler MF. 2021. The cvn8 Conservon System Is a Global Regulator of Specialized Metabolism in Streptomyces coelicolor during Interspecies Interactions. mSystems 6:e0028121.

5.	Bemis KD, Harry A, Eberlin LS, Ferreira C, van de Ven SM, Mallick P, Stolowitz M, Vitek O. 2015. Cardinal: an R package for statistical analysis of mass spectrometry-based imaging experiments. Bioinformatics 31:2418–20.

6.	Pessotti R de C, Hansen BL, Reaso JN, Ceja-Navarro JA, El-Hifnawi L, Brodie EL, Traxler MF. 2021. Multiple lineages of streptomyces produce antimicrobials within passalid beetle galleries across eastern north america. Elife 10.

7.	Heuckeroth S, Damiani T, Smirnov A, Mokshyna O, Brungs C, Korf A, Smith JD, Stincone P, Dreolin N, Nothias L-F, Hyötyläinen T, Orešič M, Karst U, Dorrestein PC, Petras D, Du X, van der Hooft JJJ, Schmid R, Pluskal T. 2024. Reproducible mass spectrometry data processing and compound annotation in MZmine 3. Nat Protoc 19:2597–2641.

8.	Kolde R. 2010. pheatmap: Pretty Heatmaps. CRAN: Contributed Packages https://doi.org/10.32614/CRAN.package.pheatmap.

9.	Petras D, Phelan V V, Acharya D, Allen AE, Aron AT, Bandeira N, Bowen BP, Belle-Oudry D, Boecker S, Cummings DA, Deutsch JM, Fahy E, Garg N, Gregor R, Handelsman J, Navarro-Hoyos M, Jarmusch AK, Jarmusch SA, Louie K, Maloney KN, Marty MT, Meijler MM, Mizrahi I, Neve RL, Northen TR, Molina-Santiago C, Panitchpakdi M, Pullman B, Puri AW, Schmid R, Subramaniam S, Thukral M, Vasquez-Castro F, Dorrestein PC, Wang M. 2022. GNPS Dashboard: collaborative exploration of mass spectrometry data in the web browser. Nat Methods 19:134–136.

10.	Seyedsayamdost MR, Traxler MF, Zheng S-L, Kolter R, Clardy J. 2011. Structure and biosynthesis of amychelin, an unusual mixed-ligand siderophore from Amycolatopsis sp. AA4. J Am Chem Soc 133:11434–7.

11.	Su L, Souaibou Y, Hôtel L, Paris C, Weissman KJ, Aigle B. 2024. Biosynthesis of novel desferrioxamine derivatives requires unprecedented crosstalk between separate NRPS-independent siderophore pathways. Appl Environ Microbiol 90:e0211523.

12.	Chen K, Rannulu NS, Cai Y, Lane P, Liebl AL, Rees BB, Corre C, Challis GL, Cole RB. 2008. Unusual odd-electron fragments from even-electron protonated prodiginine precursors using positive-ion electrospray tandem mass spectrometry. J Am Soc Mass Spectrom 19:1856–66.
